# Volatile Composition and Sensory Profiles of a Shiraz Wine Product Made with Pre- and Post-Fermentation Additions of *Ganoderma lucidum* Extract

**DOI:** 10.3390/foods8110538

**Published:** 2019-11-01

**Authors:** Anh N.H. Nguyen, Dimitra L. Capone, Trent E. Johnson, David W. Jeffery, Lukas Danner, Susan E.P. Bastian

**Affiliations:** 1 School of Agriculture, Food and Wine, The University of Adelaide, Waite Campus, PMB 1, Glen Osmond, SA 5064, Australiadimitra.capone@adelaide.edu.au (D.L.C.); trent.johnson@adelaide.edu.au (T.E.J.); david.jeffery@adelaide.edu.au (D.W.J.); lukas.danner@adelaide.edu.au (L.D.); 2 Australian Research Council Training Centre for Innovative Wine Production, The University of Adelaide, Waite Campus, PMB 1, Glen Osmond, SA 5064, Australia

**Keywords:** sensory analyses, rate-all-that-apply (RATA), headspace solid-phase micro-extraction (HS-SPME), gas chromatography‒mass spectrometry (GC-MS), wine volatiles

## Abstract

Novel Shiraz red wine products enriched with *Ganoderma lucidum* (*GL*) extract, a traditional Asian medicinal mushroom, were developed and characterized. *GL* extract was added at different levels prior to and after primary fermentation to investigate its impact on the juice fermentation kinetics, and the chemical composition and sensory properties of the resulting wines. The fermentation kinetics of red grape juice were not significantly different between ferments. Basic chemical analyses plus headspace solid-phase micro-extraction (HS-SPME), gas chromatography‒mass spectrometry (GC-MS), and a rate-all-that-apply (RATA) (*n* = 65) sensory panel were used to investigate the influence of *GL* extract additions on wine composition and sensory characteristics. Of the 54 sensory attributes assessed, 39 significantly differentiated the wines. A clear separation between *GL* wine treatments was evident with PLS regression, where specific volatiles were correlated with relevant sensory attributes that dominated the wines. These products could be promising for emerging wine markets.

## 1. Introduction

*Ganoderma lucidum* (*GL*) is an edible mushroom that has been used in Traditional Chinese Medicine for thousands of years, owing to a belief in its ability to lower cancer risk and the incidence of heart disease, as well as enhance the human immune system [1,2]. In the past, the mushroom was scarce in the wild, and was revered and served as a special food or tea that was believed to prolong the human lifespan due to its nutritional composition [3]. Recently, commercial cultivation has started and *GL* has become readily available on the market [4].

Pharmacological and clinical trials have demonstrated that *GL* can offer a wide range of medicinal benefits [3,5,6,7]. With the advent of modern science and technology, *GL* has now become a universal biological ingredient found in pharmaceutical powders and capsules [8], dietary supplements [1], and compounded medicines [9]. Previous research has not only reported the positive health effects of *GL’s* bioactive compounds such as the triterpene acids and polysaccharides [10], but also highlighted the successful inclusion of *GL* in a wide variety of foods and beverages [2,4,5]. This explains why *GL* has drawn a large amount of attention from numerous groups working on the research and development of *GL* functional foods and beverages.

Interestingly, several forms of *GL*, including fruiting body powder or extract and mycelia, have been used to produce functional *GL* foods and beverages. *GL* mycelia have been fermented on different substrates for tea production [11] and soy milk fermentation [12]. Additionally, Kim et al. [13] applied *GL* extract during the process of alcoholic fermentation to enhance the functional properties of Korean rice wine (Yakju). Other functional beverages with health-promoting properties have also been produced with added *GL* to improve the perceived body (for example, in a Serbian Pilsner beer [4]) and the sensory properties of grape brandy/distillate wine [14]. Both Leskosek-Cukalovic et al. [4] and Pecić et al. [14] added *GL* extract as a raw material when developing their products: the former aseptically added *GL* extract to commercial Pilsner beer, whereas the latter cut *GL* fruiting bodies into pieces (1 cm) and subsequently mixed them with local homemade grape brandy and wine distillate (40% *v/v*). However, neither of these studies investigated whether the presence of *GL* extract impacted the fermentation process, and the kinetics of alcoholic fermentation with *GL* additions have not been well documented.

Although there have been several studies on developing new *GL-*based foods and beverages, there appear to be no reports related to grape wine. Nguyen et al. [15] reported that most consumers in a three-nation study (Australia, China, and Vietnam) had positive attitudes toward *GL* wine products. As such, there is potential demand for these types of products in specific markets such as in Asia, where most consumers are more likely to be familiar with and have a strong belief in the potential health benefits of *GL*. However, a detailed assessment of the chemical composition, including volatiles and sensory attributes such as color, aroma, taste, and flavor of novel *GL-*based products, would need to be undertaken to assess their market potential. To our knowledge, there has been only one study that has examined *GL* extract in an alcoholic beverage fermentation [13]. These researchers demonstrated that the fermentation of a Yakju rice wine with *GL* extract mixed in the rice mash improved the consumer acceptability of the product. However, the volatile chemicals were not measured in this wine, and the sensory analyses were limited. To date, no one has performed an in-depth examination of the sensory profiles and chemical composition of foods or beverages containing *GL*.

This study was conducted to address: (1) the knowledge gap about the impact of *GL* on wine fermentation and (2) the lack of detailed sensory and chemical profiles of foods made with *GL*, with the ultimate aim of exploring the potential of producing a new wine product containing *GL* for the Asian markets. To achieve this, the effect of *GL* addition on the progression and completion of Shiraz red wine primary fermentation was evaluated. Additionally, the differences in chemical composition and sensory profiles between wines made with different levels of *GL* extract added either during or after fermentation were assessed using; basic wine chemical measures, volatile chemical analyses by headspace solid-phase micro-extraction (HS-SPME) coupled with gas chromatography‒mass spectrometry (GC-MS), and the rate-all-that-apply (RATA) [16] sensory methodology. Correlations between significant chemical and sensory data were made using partial least squares (PLS) regression to understand the chemical drivers of the perceived sensory attributes identified in the trial wines.

## 2. Materials and Methods

### 2.1. Study Design

This study consisted of three distinct parts: (1) preliminary experimental small-scale (100 mL) fermentations in chemically defined media and Shiraz grape juice to determine the concentrations of *GL* extract that did not impact on fermentation kinetics and inform the next phase; (2) medium-scale fermentations of Shiraz juice (5 L), which together with commercial wine were evaluated by two preliminary benchtop sensory panels using check-all-that-apply (CATA) to determine the *GL* concentrations suitable for fermentation and sensorial acceptability of wines in (3) where larger-scale winemaking (28 L) was conducted to produce a sufficient number of wine samples that were subsequently used in a formal descriptive sensory test (RATA) and detailed chemical analyses, allowing for the examination of relationships between the sensory characteristics and chemical components of *GL* wine. Based on the Australian and New Zealand Food Standards (Standard 2.7.4), any such wine would be considered a “wine product,” but will be referred to as wine throughout the remainder of the text for simplicity.

### 2.2. GL Extract

*GL* extract powder (dual alcohol and triple hot water extracted, 1 kg) was purchased from the Super Food Australia Company (Blackheath, New South Wales, Australia) and stored at room temperature (approximately 23 °C).

### 2.3. Fermentation

*Small-scale fermentations (100 mL)*. Ten grape juice *GL* extract mixtures (100 mL) were produced in triplicate by adding extracts at five different levels (0, 4.5, 9, 18, and 36 g/L) into 100 mL of chemically defined grape juice media (CDGJM) [17] and 100 mL of cross-flow filtered and cold stabilized 2016 Australian Shiraz red grape juice (RGJ) purchased from Patritti Wines (Dover Gardens, Adelaide, SA, Australia). Fermentations were conducted using 250-mL Erlenmeyer flasks fitted with airlocks, as described in a previous study [18]. The strain of *Saccharomyces cerevisiae* (*S. cerevisiae*) used was the commercial wine yeast EC1118 (300 mg/L) (Lallemand, Edwardstown, SA, Australia), which was rehydrated and grown in yeast extract, peptone, and dextrose media (YEPD), consisting of 1% yeast extract (Amyl Media, Dandenong, VIC, Australia), 2% bacteriological peptone (Amyl Media, Dandenong, VIC, Australia), and 2% glucose (Chem-Supply, Gillman, SA, Australia). Fermentation was monitored by measuring the °Brix values of each ferment daily, using a PAL-1 portable refractometer (Atago, Tokyo, Japan) until 6 °Brix between seven and 10 days when ferments had plateaued and were terminated [17]. °Brix values measured in the small-scale fermentation are shown in Table S1.

*Medium-scale fermentations (5 L)*. To determine the acceptable organoleptic levels of *GL* in wine for sensory analyses in the larger-scale fermentations, mixtures of Shiraz grape juice (Patritti Inc., 13-23 Clacton Rd, Dover Gardens 5048, SA, Australia) and *GL* extract were prepared at different levels of *GL* based on the 100 mL experiments. The *GL* concentrations trialed were 0, 4.5, and 9 g/L in 5 L of juice, with the fermentation being conducted using the same protocol as the small-scale fermentations described above.

The resultant three medium-scale wines were assessed by a sensory panel (*n* = 11 participants, who were either University of Adelaide students enrolled in postgraduate coursework oenology and viticulture programs or higher degree research students aged between 28 and 35 years) using a CATA analysis. Additionally, a second preliminary evaluation of a commercially available South Australian 2016 Shiraz wine (Yalumba, Angaston, Australia; alcohol: 14% *v*/*v*) used as a base wine and enriched with different amounts of *GL* (0, 2.25, 4.5, 6.75, and 9 g/L, added immediately before the benchtop tasting occurred), were examined by a sensory panel (*n* = 32 University of Adelaide students enrolled in postgraduate coursework oenology and viticulture programs or higher degree research students aged 28 and 35 years). For both CATA panels, 30 mL of each wine were assigned a random three-digit code and presented in transparent ISO-standard glasses, in randomized order for blind tasting for liking, intensity ratings of specific attributes, and CATA analysis. In the tasting session, participants were first asked to rate their wine liking on a nine-point scale (1 = dislike extremely, 5 = neither like nor dislike, 9 = like extremely) and rate the intensity of seven sensory attributes (aroma, sweetness, acidity, hotness, umami, bitterness, and astringency) on a seven-point scale (1 = extremely low, 4 = moderate intensity, 7 = extremely high) for each wine. The last part of the sensory session involved the CATA, where participants only ticked aroma or flavor attributes that they perceived to be present in the wine based on an attribute list (tropical, lychee, citrus, red berry, cherry, dark berry, dried fruit, jammy, confectionery, floral, honey, herbaceous, oak, sweet oak, leather, tobacco, spice, pepper, earthy, mushroom, and savory notes) generated by an expert benchtop trial with five wine academics. Panelists individually rated each wine in an open-plan sensory facility and took a 1-min forced break between each wine, and had access to water and plain crackers as palate cleansers. Data were collected by paper ballot.

Preliminary CATA analysis of the commercial Shiraz wines with added *GL* (Table S2) showed that the majority of sensory attributes were not significantly different between wines, with the exception of earthy and mushroom aromas, which were noted significantly more in *GL*-treated wines and oak, which was significantly lower in *GL*-treated wines than in the control wines. In the medium-scale fermentation wines, significantly higher floral, tropical, and lychee flavors were found in the control wine (Table S2).

Regarding the intensity of aroma and palate attributes, increasing the levels of *GL* addition (up to 9 g/L) in commercial Shiraz wines did not significantly impact the intensities of acidity, heat, umami, and astringency, but the aroma intensity and bitterness were significantly higher in the 9 g/L *GL* wine (Table S2). Sweetness differences were not as clear, but the 4.5 g/L wine was significantly sweeter than the 6.75 g/L wine. In the case of extracts added during the medium-scale fermentation, *GL* wines were perceived as significantly less sweet, more acidic, hotter, and more bitter than the control wines (Table S2).

*Larger-scale fermentations (28 L).* Juice (500 L) was sourced from Patritti Inc. (2017 Australian Shiraz; 22 °Brix, pH 3.4; 3.9 g/L titratable acidity (TA)). Before fermentation commenced, the juice was dispensed into 28 L batches in sterilized (hot water and 70% ethanol-washed) plastic drums (30 L) and stored frozen at −15 °C until required (the fermentation process flow diagram is shown in Figure S1). The frozen juice was thawed for two days at room temperature, then mixed with different concentrations of *GL* extracts, or fermented and then mixed with *GL* extracts. Treatments conducted in triplicate comprised *GL* added before fermentation at 1 g/L (PRE 1a, b and c), 2 g/L (PRE 2a, b and c), and 4 g/L (PRE 4a, b and c), and after fermentation (at bottling) at 1 g/L (POST 1a, b and c) and 4 g/L (POST 4a, b and c) eventually resulting in a total of 18 wines. Juice treatments were thoroughly mixed to ensure the liquid and extract were fully homogenized before fermentation. The concentrations of *GL* extract added into the juice before and after fermentation were determined by a literature review of other foods and beverages supplemented with *GL* [13] and from the preliminary CATA sensory experiment results (Table S2), which showed that the wines were neither liked nor disliked at added *GL* levels ranging from 0 to 6.75 g/L, while the wines with 9 g/L were not liked and were perceived as significantly hotter and more bitter than the wines with lower levels of *GL* addition. Therefore, the concentrations of *GL* applied in 28 L winemaking were 0, 1, 2, and 4 g/L at different stages of the fermentation process. Control wines (control a, b, and c) were fermented under the same fermentation conditions, but without extract addition. After the addition of 100 mg/L diammonium phosphate (DAP), the juice was inoculated with 300 mg/L of Lalvin EC1118 yeast (Lallemand) and co-inoculated after two days with 100 mg/L of Lalvin VP41 malolactic bacteria—*Oenococcus oeni* (Lallemand). Alcoholic fermentations were performed in a temperature-controlled room at 17 °C. During alcoholic fermentation, the °Brix of each fermenter was monitored daily using a density meter (Anton Paar DMA 35, Graz, Austria) until approximately 2 °Brix. Dryness (i.e., residual sugar (RS) < 2 g/L glucose and fructose) was determined enzymatically with a K-FRUGL test kit (Megazyme, Wicklow, Ireland), internally calibrated using 4 calibrators of 0, 0.75, 1.5, and 3.0 g/L of each sugar (d-(-)-fructose and d-(+)-glucose (Sigma, St. Louis, MO, USA)). Malolactic fermentation (MLF) was considered complete when the malic acid levels were in the range of 0.1–0.4 g/L (l-malic acid enzymatic test kit, Vintessential Laboratories, Dromana, VIC, Australia). After malolactic fermentation, wines were racked off gross lees, 60 mg/L of potassium metabisulfite (PMS) was added as an aqueous solution (10% *w*/*v*), and wines were cold-stabilized at 0 °C for 21 days.

After stabilization, PMS was added to yield free SO_2_ levels of 40–50 mg/L before bottling. Additions of *GL* extracts to wine after fermentation at either 1 g/L or 4 g/L were made with stirring just before bottling. Wines were bottled by WIC Winemaking Services (The University of Adelaide, Urrbrae, Australia) in 750-mL green Bordeaux-shaped bottles closed with aluminum screw caps (Stelvin caps) under nitrogen gas using a Framax filling system (Serravalle Pistoiese, Pistoia, Italy) and Arol closure system (Costa Enterprises, Canelli, Italy). Bottled wines were stored in a temperature-controlled room at 15 °C for three months and equilibrated at room temperature (22‒23 °C) before sensory analyses and sampling for future chemical analyses.

### 2.4. Basic Chemical Analyses

Basic juice and wine composition measurements of the larger-scale wines were performed in triplicate, while volatile acidity (VA) measurements were conducted in duplicate. °Brix values were measured in juice using a portable density meter (Anton Paar DMA 35). Free and total SO_2_ content and VA in juice and wine were determined using the methods described in previous studies [19]. Measurements of pH and TA, color (CIELAB), and ethanol content (% *v*/*v*) were undertaken with a T50 Titrator (Mettler-Toledo, Port Melbourne, Australia), Cintra 4040 (GBC Scientific Equipment, Victoria, Australia), and Alcolyzer ME/DMA 4500 M (Anton Paar), respectively. Yeast assimilable nitrogen was determined enzymatically with a Chemwell 2910 auto-analyzer (following the procedure for K-PANOPA and K-AMIAR kits (Megazyme, Wicklow, Ireland)).

### 2.5. Headspace Solid-Phase Micro-Extraction (HS-SPME-GC-MS)

For quantitative analyses of the major volatile compounds in the headspace of the larger-scale wines, samples were prepared, extracted, and analyzed according to the method described in a previous study [20]. Analyses were undertaken with a Gerstel MPS auto-sampler (Lasersan Australia Pty, Ltd., Robina, Australia), coupled with an Agilent 7890A gas chromatograph (Agilent, Palo Alto, CA, USA) and combined with an Agilent 5975C mass selective detector (Agilent). Separations were performed with a DB-Waxetr column (60 m, 0.25 mm i.d., 0.25 μm film thickness, Agilent J&W, Folsom, CA, USA) with carrier gas at a constant flow rate of 2 mL/min. All other instrument parameters were as previously specified [20].

### 2.6. Rate-All-That-Apply Sensory Evaluation of GL Wines

RATA is a rapid sensory method that can use trained panelists or untrained wine consumers to objectively generate sensory profiles of wine, requiring less time and cost than traditional profiling methods such as descriptive analyses (DA). Studies have demonstrated that the RATA sensory profiles generated for multiple sets of wines were comparable to those produced by a DA panel [16] and have been successfully utilized with consumers to profile unfamiliar wines [21].

Regular red wine drinkers (*n* = 65, aged between 28 and 35 years, 50.7% female) from among postgraduate coursework oenology and viticulture programs and higher-degree research students enrolled at the University of Adelaide were recruited as volunteer panelists to profile the 28 L ferment wines. This study was approved by the Human Research Ethics Committee of the University of Adelaide (Approval No. H-2016-194).

Before the formal RATA sessions, a panel consisting of five wine experts assessed the 18 wines for any faults and decided upon the addition of extra aroma or flavor attribute terms to the generic red wine RATA attribute list described in previous studies [16,22]. Added attribute terms included mushroom, earthy, and tobacco, with the ultimate sensory attribute list consisting of 23 aroma, 21 flavor, and 5 mouthfeel attributes. The sensory panel attended one 40-min formal session per week for two weeks, with nine wines presented at each session. Evaluations were conducted in individual sensory booths at 23 °C. Each wine (30 mL) was presented in transparent ISO-standard wine glasses, labeled with three-digit-codes, and covered with glass Petri dishes at room temperature (23 °C). Wines were served sequentially and monadically in a randomized order, balanced for carryover effects [23]. Panelists assessed the wine samples after smelling (for aroma assessments) and tasting (for flavor assessments), and only rated the intensity of each sensory attribute that they perceived to be present on the line scale, as described in a previous study [16]. A rest of 1-min between samples and a 5-min break after the first four samples was enforced, and water and crackers were provided for palate cleansing.

### 2.7. Data Analyses

Basic chemical data were analyzed by a one-way analysis of variance (ANOVA) with Tukey’s HSD post hoc test using SPSS 23 (IBM Corporation, Armonk, NY, USA). The Cochran’s Q test was used to compare the impact of a wide range of *GL* levels on the sensory characteristic of sample wines in the CATA testing. RedJade (Redwood City, CA, USA) online software was used to collect the sensory data generated in the RATA testing. SENPAQ (version 5.01, Qi statistic, Ruscombe, UK) was used to identify the sensory attributes that significantly differentiated the wine samples, using two-way analysis of variance (ANOVA) with participants as random and samples as fixed factors. Fisher’s LSD was used for post hoc comparisons. Significant sensory attribute means were subjected to principal component analyses (PCA) using XLSTAT (version 2018, Addinsoft, New York, NY, USA). Volatiles were analyzed by one-way ANOVA using XLSTAT, and all significantly different sensory attribute means and chemical components were subjected to PLS regression analyses along with basic chemical components using The Unscrambler (version 9.7, CAMO software A, Oslo, Norway). All statistical tests were conducted using a significance level of 0.05.

## 3. Results and Discussion

### 3.1. Impact of GL Concentrations on Fermentation Kinetics

#### 3.1.1. Small-Scale Fermentations

*GL* extract has been shown to have antimicrobial effects [24], so it was necessary to evaluate the impact on yeast by the addition of extracts prior to the fermentation of grape juice by monitoring the changes in sugar content in the must. Initially, small-scale fermentations of either RGJ or CDGJM containing *GL* extract added at different concentrations (0, 4.5, 9, 18, and 36 g/L) were conducted to determine whether the presence of *GL* extract in the juices impacted the kinetics of fermentation. At the beginning of the fermentation, the initial °Brix values were moderately different between the treatments due to the variable extract levels added before fermentation, whereby extracts influenced the refractometer measurements (Table S1). However, the range of means of °Brix value at the beginning on day 0 (25.9 − 23.6 = 2.3 for the RGJ and 24.3 − 21.6 = 2.7 for CDGJM) were similar to those of the samples at the end of fermentation (10.7 − 8.2 = 2.5 for RGJ and 9.8 − 6.6 = 3.2 for CDGJM) (Table S1), which indicated that the fermentation kinetics behavior was similar between the two juices as most of the sugars in each ferment were metabolized in the same time period. However, fermentation was slightly slower for control ferments without *GL* extract between days 2 and 4. Minimal inhibitory concentrations of *GL* extracts recorded in previous studies were 0.0125−1.25 mg/mL [24,25]; therefore, the *GL* levels applied in the small-scale fermentations did not appear to have inhibited the yeast performance in the fermentation process. The presence of a variety of sugars and other metabolites from *GL* extract added at 36 g/L or lower in the juice in the current study did not appear to hamper the wine fermentation in either RGJ or CDGJM (Table S1), but were possibly slightly different to control. It is suggested that further investigations need to be done with high-performance liquid chromatography (HPLC) or enzymatic assays on wines fermented with *GL* to comprehend the residual sugar profile after fermentation, including the polysaccharides noted in previous studies [1,9,26,27]. Future studies could examine the yeast metabolism of *GL* extract to have a better understanding of whether they are able to digest constituents other than sugars originating from *GL* extract.

#### 3.1.2. Larger-Scale Fermentations

**Figure 1:**
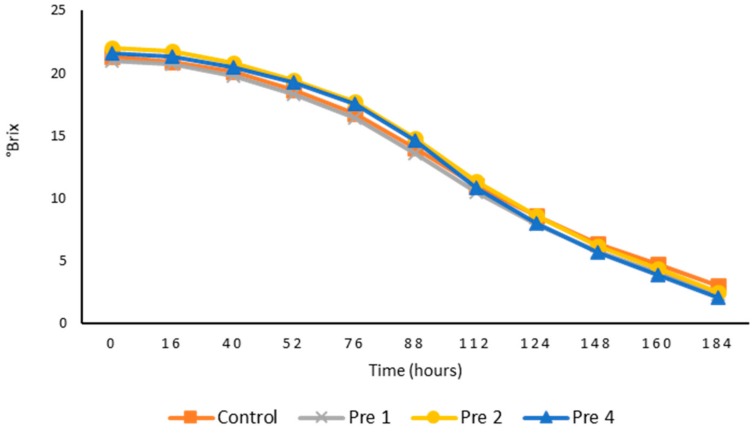
Comparative mean °Brix values of triplicate, larger-scale 28 L ferments of control juice and juice with different *GL* additions.

Fermentation kinetic behavior of the 28 L wine ferments without (control) and with the presence of *GL* at different levels (1, 2, and 4 g/L) was consistent between samples from the beginning to the end of fermentation (Figure 1). Enzymatic measurements of the residual sugars in these wines were less than or equal to 2 g/L, and therefore the wines were considered to be dry. This indicated that the fermentation process had finished successfully, and the addition of *GL* did not impact on the ability of yeast to undertake alcoholic fermentation. Furthermore, the presence of *GL* extract did not impede the MLF, as all wines contained malic acid levels below 0.4 g/L. 

### 3.2. Sensory and Chemistry Profiles of GL Wines

**Figure 2:**
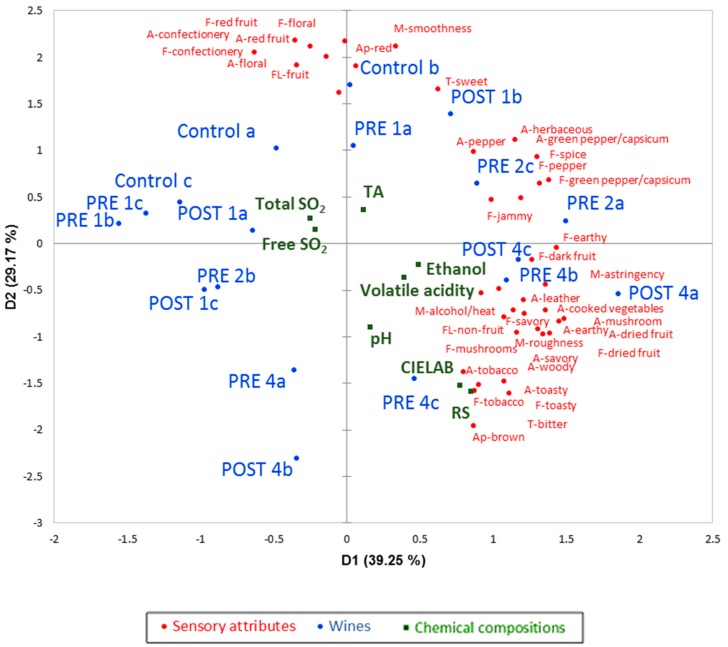
PCA biplot of mean sensory data obtained from the RATA panel (*n* = 65) overlaid with basic chemical analyses as supplementary data for 18 wines (28 L ferments) made with and without *GL* extracts added pre- and post-fermentation. Prefix A- = aroma attribute, T- = taste, F- = flavor attribute, M- = mouthfeel, Ap- = appearance, FL- = aftertaste (fruit and nonfruit). Prefixes PRE = *GL* extracts added prior to fermentation (PRE 1, PRE 2, and PRE 4), POST = *GL* extracts added after fermentation process (POST 1 and POST 4).

Compared to the controls, *GL* additions had a significant influence on the perceived red wine sensory attributes. Out of a possible 54 sensory attributes evaluated by the RATA panel, 39 were perceived to be significantly different between the treatments and related to the levels of *GL* addition (*p* < 0.05) (Table S3). The PCA of the mean intensity ratings for statistically significant sensory attributes explained 68.42% of the variation in the data with the first two principal components (PC1 = 39.25% and PC2 = 29.17%, Figure 2). Woody aroma, pepper, and spice flavors; earthy, savory, dried fruit, mushroom, and green capsicum aromas and flavors; astringent and rough mouthfeel were positively loaded on PC1 of the biplot and were associated with wines POST 4a, PRE 2a and 2c, and PRE 4b and 4c. Red appearance, red fruit, confectionery, floral notes, and smooth mouthfeel were clustered positively on PC2, while brown appearance was comparatively strongly negatively loaded and linked to a number of wines including the controls and PRE 1a and POST 1b.

Wines appearing in the top right-hand quadrant possessed green capsicum, spice, and pepper aromas and flavors, mainly associated with wines made with 2 g/L *GL* extract. These wines were also perceived as sweeter in taste that, since the wines were fermented to dryness, may be caused by compounds occurring in the *GL* extract such as polysaccharides [28] and possibly triterpenoids, reported as noncariogenic intense natural sweeteners [29]. The majority of wines in the bottom right-hand quadrant were made with the pre- or post-fermentation addition of 4 g/L *GL* extract and were dominated by mushroom, woody, earthy, toasty, and savory aromas and flavors, higher astringency and roughness, and a bitter taste. Some of these aromas—tobacco, toasty, and woody, for example—are akin to the aromas found in wines aged in oak; it is interesting that *GL* wines were not oaked but could contribute to similar oaked-wine profiles, which might appeal to some consumers [22]. On the contrary, the wines located in the two left-hand quadrants were the controls and those made with pre- and post-fermentation additions of 1 g/L *GL* extract. The wines occurring in the top left-hand quadrant were described as having more red fruit, floral, and confectionery aromas and flavors, a smooth mouthfeel, and a sweet taste. With respect to appearance, wines made with the highest amount of *GL* (4 g/L) were perceived as browner in color, which aligns with the red and yellow tendencies from the CIELAB analyses of these wines. On the other hand, control wines and those with 1 g/L of added extract were more reddish in appearance, which was also evident from the color intensity and blue-green tendencies discussed below (Table 1). In addition, the L* values of wines made with 4 g/L *GL* were lower than those of wines made with 1 and 2 g/L or without *GL* (data not shown), which was supported by a previous study conducted by Pecić et al. [30], who showed that increasing the levels of *GL* in wines resulted in decreasing L* values. It can be concluded that the wines became slightly darker with an increasing amount of *GL* added.

The current study supports the previous work conducted by Pecić et al. [14], who illustrated that high levels of *GL* added to commercial brandy changed the sensory profile of the resulting products, rendering them more bitter in taste, apparently due to the bitter acids, namely *GL* triterpene acids [3]. In the same way, adding *GL* pre- and post-fermentation in this study generated a variety of wines with new profiles, and the perceived bitterness also increased with increased *GL* additions. From the preliminary benchtop trial of commercial wines spiked with *GL* extract and the medium-scale ferments, a low level of *GL* extract content in wine (2 g/L or less) was preferred and had mean consumer liking scores close to those of the control (Table S2), but 4 g/L or higher was not liked. This may mean that higher *GL* additions promote sensory attributes that sensory panelists may dislike, including the bitterness, roughness, and astringency associated with tannins, and more barnyard aroma, as reported for red wines by Bastian et al. [31] at the expense of the red fruit, floral, and confectionery characters favored by Chinese wine professionals in rosé wines [32]. Comparing Chinese and Australian consumers’ preference for red wines, Williamson et al. [33] claimed that consumers from the two countries had similar preference drivers: for instance, red fruit flavor, sweetness, and a fruity aftertaste were some of the most important sensory attributes positively associated with consumer liking; however, bitterness and strong acidity had low consumer acceptance. Understanding of Australian, Chinese, and Vietnamese consumers’ opinions towards *GL* wines was conducted in a previous study and demonstrated their acceptance and willingness to try these wine styles [15]. A preliminary liking study reported by Kim et al. [13] indicated that Korean rice wine with 1 g/L of *GL* extract was the most acceptable compared to rice wines with a higher amount of extract (2 g/L), which caused an unfavorable color and a bitter taste. This is in agreement with our preliminary CATA and hedonic tasting of the medium-scale fermentations (as discussed above), where wines with higher levels of *GL* were less liked, possibly due to the bitterness and astringency being higher in intensity, along with less floral and tropical characters. Therefore, a future study with higher numbers of consumers could be conducted to determine preferences for *GL* wines containing lower levels (e.g., 1–2 g/L) in the Australian and Asian markets.

To produce novel *GL* wines for more extensive sensory and chemical assessment, larger-scale wine ferments with *GL* additions (determined from medium-scale wine sensory analyses) were made. The addition of *GL* pre- (1, 2, and 4 g/L) and post- (1 and 4 g/L) fermentation had a significant impact on the pH, TA, ethanol, VA, and color parameters of the resultant wines (Table 1). The pH values of the wines ranged from 3.8 to 4.0, which are the usual values in accordance with other red wine studies [34,35]. The TA values ranged from 4.1 to 4.7 (g/L), which is slightly lower than the typical range reported in commercial red wines, likely as a result of a lack of skin contact prior to or during the fermentation [36]. As alcohol concentrations ranged from 12.3 to 13.8 (% *v*/*v*) (with a mean value of 13.02% *v*/*v*), these preliminary *GL* wines would be categorized as table wines [37]. The largest difference in % *v*/*v* ethanol between *GL* wines was greater than the recently reported best estimate retronasal and orthonasal difference thresholds measured in a Zinfandel red wine [38]. However, the outcomes from RATA (Figure 2) of the *GL* wines showed that most tasters could only perceive PRE 1b as having a significantly less hot mouthfeel than all other wines.

Wine color is one of the most important wine quality factors as it impacts sensory assessments and plays a vital role in the decision-making of consumers preferring deeply-colored red wine [39]. The pre- and post-fermentation addition of *GL* extract impacted wine color (Table 1). As a good representation of human color perception, CIELAB measures were used to assess wine color, where L* represents lightness (data not shown), and a* and b* represent the extent of green‒red and blue‒yellow color, respectively [39]. Chroma C* values ranged from 10.4 to 14.7, with higher color values observed with greater additions of *GL,* which is in agreement with previous studies [13,30]. Our results suggest that the *GL* wines would be noticeably different in color ΔE*_ab_ between low (1 g/L, more red appearance) and high (4 g/L, more brown appearance) level treatments (ΔE*_ab_ = 2 and 3 CIELAB units for pre- and post-treatment, respectively), which was consistent with the RATA data (Figure 2) that determined wine color significantly differentiated samples. Furthermore, the wines produced with the *GL* extract added prior to fermentation had not only a significantly lower calculated color intensity, but also lower a* and b* values (1 g/L: a* = 10.1, b* = 5.8; 4 g/L: a* = 9.5, b* = 8.1) compared to wines with the same level of *GL* extract added after fermentation (1 g/L: a* = 10.3 and b* = 7.2; 4 g/L: a* = 11.3 and b* = 9.1). The RATA sensory test indicated that the 4 g/L wines were perceived to be deeper in color (L* = 83) than control wines and wines with 1 g/L *GL* addition (L* ranged from 85 to 88, respectively), as the L* value reduced along with an increasing *GL*. These findings confirm that the RATA panel results were consistent with the CIELAB measures, indicating that the panel was performing to a high level. Bisson [40] reported that wines are defined as dry when their RS values are less than 4 g/L at the end of the alcoholic fermentation. The range of RS observed in this study was between 0.61 and 2.96 g/L, consistent with the RS level of dry red wines [37], meaning they would not be perceptibly sweet due to grape-derived glucose and fructose.

To date, only a few studies have examined the volatile compounds from *GL* mycelia by HS-SPME-GC-MS (the most abundant being 1-octen-3-ol, ethanol, hexanal, 1-hexanol, sesquirosefuran, 3-octanol, and 3-octanone) [41] and from *GL* fruiting body (the major occurring compounds being 1-octen-3-ol, 1-octanol, and 3-methyl butanal) using HS-SPME-GC-MS [42]. However, no research has investigated the relationships between the sensory characteristics and chemical components of foods and beverages made with *GL* extract. Therefore, the next step of the study examined the correlations between the chemical composition and sensory profiles of the *GL* wine samples and permitted further examination of the impact of adding *GL* extract either pre- or post-primary alcoholic fermentation.

### 3.3. Volatile Compounds in GL Wine

Trial wines underwent HS-SPME-GC-MS analyses to evaluate a range of volatiles (Table 2). Among the 29 volatile compounds quantified across the treatments, ethyl and acetate esters were the most abundant. These are fermentation-derived compounds and are known to be responsible for fruity and floral notes in wine [43,44]. Furthermore, eight volatile compounds, including 2-phenylethanol and 1-octanol, ethyl acetate, limonene, and hexanoic, octanoic, decanoic, and 3-methylbutanoic acids, were found to differ significantly between the treatments (bolded significant values in Table 2). Nine odorants occurred in wine samples at concentrations higher than their reported odor detection thresholds, including ethyl butanoate, 1-propanol, 3-methylbutyl acetate, ethyl hexanoate, nonanal, 3-methylbutanoic acid, β-damascenone, hexanoic acid, and octanoic acid. In particular, 3-methylbutyl acetate, which contributes to fruity (banana, pear) characteristics, was present at a concentration of 577 μg/L, 16 times higher than the reported odor detection threshold that was reported in other research regarding the odor detection thresholds of these specific volatiles [20,45,46]. Notably, the concentration of β-damascenone was 24 times above the reported odor threshold, emphasizing its possible important contribution to fruity flavors (apple, rose, honey, candy, and citrus) in these Shiraz wine products, which is in agreement with other studies [47,48].

### 3.4. Correlation between Chemical and Sensory Data of GL Wines

**Figure 3:**
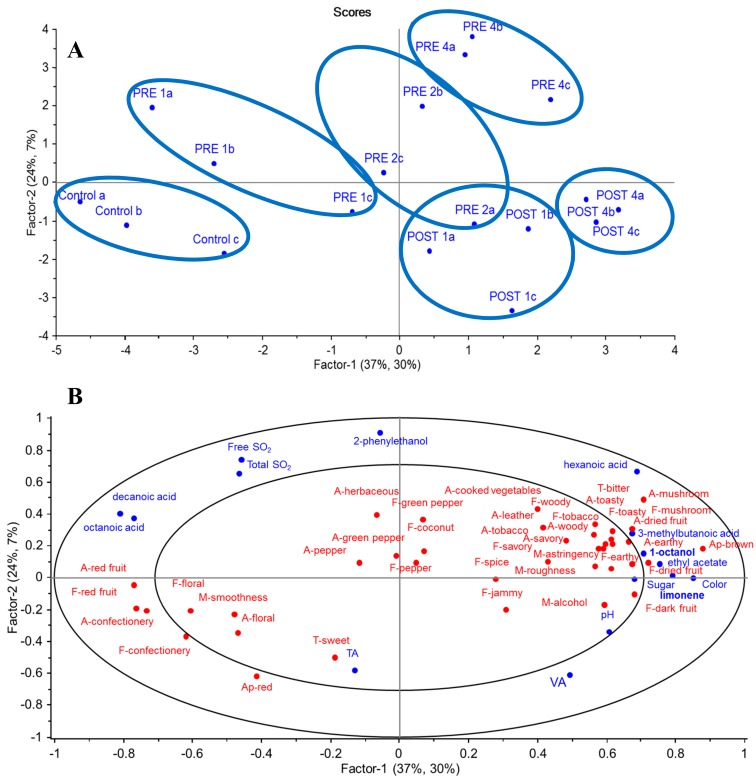
PLS regression and scores plots of significant volatile compounds (*p* < 0.05), sensory attributes (*p* < 0.05) and basic chemical data for 18 wines (28 L ferments) made with and without *GL* extracts added pre- or post-fermentation. X-variables: chemical components, Y-variables: sensory descriptors. The inner and outer ellipses represent *R^2^* = 50% and 100%, respectively. (**A**) sample configuration, prefix PRE = *GL* extracts added prior to fermentation (PRE 1, PRE 2 and PRE 4) and POST = *GL* extracts added after the fermentation process (POST 1 and POST 4). (**B**) attribute configuration with prefix A- = aroma attribute; T- = taste, F- = flavor attribute, M- = mouthfeel, Ap- = appearance, FL- = aftertaste (fruit and nonfruit). TA = titratable acidity, VA = volatile acidity.

To explore the underlying relationships between wine chemistry and sensory data, significantly different data for volatile compounds, basic chemical components, and RATA sensory attributes were subjected to PLS (Figure 3). Figure 3A shows that there was a relatively clear separation between wine treatment groups from the PLS scores plot, where the first two factors explained 61% of the variation in wine chemical composition (x-variables) and 37% of the variation in sensory attributes (y-variables). Wines made with *GL* additions before fermentation (PRE) were primarily located in the two top left and right quadrants of the plot (transitioning from lower to higher additions rates, going from left to right), whereas wines made without *GL* (control) and with *GL* supplemented post-fermentation (POST) appeared in the bottom left and right quadrants, respectively (Figure 3A). In Figure 3B, the first factor (x explained 37% of total variance in chemical composition, y explained 30% of total variance in sensory characteristics) distinguished wine samples on the left side of the plot mainly according to red appearance, red fruit, and confectionery notes, compared to the right side of the plot, which contained brown appearance; spicy, jammy, earthy, and dark fruit flavors; rough, hot mouthfeel; and bitter taste. The second factor (x explained 24% of the total variance in chemical composition, y explained 7% of the total variance in sensory characteristics) separated samples vertically, from the bottom section with a sweet taste to the top section, which was mainly dominated by an herbaceous aroma and green capsicum flavor.

Wine aroma attributes located in the left quadrants, such as red fruit, confectionery, and floral characteristics, were positively correlated with volatile acids (decanoic acid and octanoic acid) and negatively correlated with 1-octanol, 3-methylbutanoic and hexanoic acids, limonene, and ethyl acetate (Figure S2A,B). The study findings were supported by a previous study conducted by Vilanova et al. [44], who determined that decanoic acid and octanoic acid are typically associated with fruit attributes (ripe fruity attributes).

Sensory attributes on the right quadrant such as woody, dried fruit, earthy, and mushroom notes and bitter taste were positively correlated with hexanoic acid, 1-octanol, and limonene. Furthermore, hexanoic acid is known for its leafy, wood descriptors [20], while limonene, derived from grapes, relates to floral and citrus (lemon and orange) characteristics in wines [47,49]. Interestingly, Robinson et al. [50], who used PLS regression to predict the relationship between sensory attributes and chemical composition, indicated that both 1-octanol and limonene were correlated with the bitter taste of Cabernet Sauvignon red wines, which aligns with our study findings. In Figure 3, it is noteworthy that a positive correlation was found between 1-octanol and dried fruit (Figure S2C,D), in agreement with a previous study [50]. Similarly, there was a positive correlation between 1-octanol and mushroom notes (Figure S2E,F). Taskin et al. [42] determined that there were 18 aroma compounds found in *GL*’s mycelia including 1-octanol, which implies the 1-octanol in our *GL* wines (4 g/L) could be derived from *GL* extract.

Pre-fermentation *GL* wines appearing in the top section displayed peppery, coconut, green capsicum, and herbaceous attributes, showing a positive correlation with 2-phenylethanol, which imparts floral and rose attributes [47]. On the contrary, wines with *GL* added after fermentation were mainly located in the bottom section, representing less cooked vegetable aroma, a mushroom or leather aroma, green capsicum, toasty, and tobacco flavor, and a less bitter taste.

Considering the potential for producing a *GL*-based wine product [15], various studies were undertaken to investigate aspects of fermentation, chemical composition, and sensory profiles. *GL* extract addition to juice or chemically defined grape juice media was deemed not to overly affect fermentation, with the pattern of sugar consumption being very similar for all the treatments. On the other hand, *GL* extract addition does impact on the sensory and chemical components of the resultant wines. It can be concluded that the higher the concentration of *GL* used in treatments, the more bitter the wines tasted due to the bitterness derived from the triterpenes in the *GL* extract added [30]. Of note, limonene and 1-octanol volatile compounds significantly differentiated between wines and were positively related to wines’ bitterness and dried fruit flavor (Figure 3), in accordance with a previous study [50]. It may be possible to remove these bitter compounds with the use of fining agents commonly used in the wine industry, or at least to suppress the bitter taste with the retention of low amounts of residual sugar by not fermenting wines to dryness. The aromatic profile also changed with the highest *GL* addition, with these wines being more complex and having more dark and dried fruit, toasty, earthy, and woody notes. Thus, it is likely that a couple of wine styles could be produced that may potentially suit different consumer segments’ preferences.

## 4. Conclusions

This work is the first to report on the impact of *GL* on the primary alcoholic and secondary malolactic fermentation of Shiraz grape wine. Based on our earlier findings that the concept of a *GL*-containing wine was acceptable to consumers from three different countries, we have employed a consumer-centric product development approach to produce prototype wines. Furthermore, we utilized novel and rapid wine sensory evaluation methods and compositional analyses to advance our knowledge of the sensory and chemical properties of Australian-made *GL*-containing Shiraz wines.

*GL* extract addition did not impact wine fermentation, but influenced the sensory profiles and chemical composition of the resulting wines. Fermentation kinetic behaviors were similar between treatments, and stuck or sluggish fermentations were not observed. Thirty-nine sensory attributes, together with eight volatile compounds, significantly differentiated the wine treatments. In addition, specific volatiles correlated with relevant sensory attributes, particularly in 4 g/L *GL* wines. For instance, 1-octanol was positively related to wines’ mushroom notes. These initial experiments on winemaking with *GL* extracts are promising and will enable winemakers to gain insight into potential new wine products, which could be of interest to Asian consumers who display strong consumer behavior in the use of *GL* and its products. Future research could focus on target market consumers’ preferences in conjunction with hedonic clustering to determine the specific sensory attributes and chemical components that may drive consumer segment liking. This will assist the wine industry in gaining a deeper understanding of consumers’ preferences for novel wines containing traditional Asian medicinal mushroom extracts designed for the Australasian market.

## Figures and Tables

**Table 1:** Basic chemical composition of the *GL* wines from 28 L ferments.

Treatment Samples	pH	Titratable Acidity (g/L)	Ethanol (% *v*/*v*)	Volatile Acidity (g/L)	Free SO_2_ (mg/L)	Total SO_2_ (mg/L)	Chroma C^*^(D650)	a* (D650)	b* (D650)	Residual Sugar (g/L)
Control a	3.91 gh	4.63 b	12.43 gh	0.25 f	48.53 f	112.53 j	10.49 m	8.89 k	5.58 m	0.68 hi
Control b	3.91 gh	4.56 c	12.75 f	0.25 f	48.53 f	123.73 fg	10.54 l	8.85 l	5.73 l	0.61 j
Control c	3.93 efg	4.73 a	12.79 ef	0.32 c	51.20 e	121.60 h	10.59 k	8.81 m	5.87 j	0.68 hi
PRE 1a	3.84 i	4.16 lm	12.91 e	0.25 f	62.40 a	134.4 a	11.70 i	10.11 f	5.89 j	0.67 ij
PRE 1b	3.90 h	4.21 jk	12.30 hi	0.25 f	51.73 de	117.33 i	11.67 j	10.16 e	5.75 l	0.74 h
PRE 1c	4.02 a	4.40 e	12.73 f	0.32 c	52.80 d	128.53 c	11.68 i	10.14 ef	5.82 k	0.67 ij
PRE 2a	3.97 b	4.58 c	13.32 c	0.39 a	48.00 f	122.13 gh	11.81 h	9.80 h	6.62 h	0.95 g
PRE 2b	3.93 efg	4.12 m	13.36 bc	0.25 f	49.06 f	124.26 ef	11.82 h	9.82 gh	6.59 hi	1.01 g
PRE 2c	3.92 efgh	4.29 hi	13.16 d	0.25 f	52.80 d	125.86 de	11.82 h	9.84 g	6.56 i	0.98 g
PRE 4a	3.92 fgh	4.18 kl	12.25 i	0.25 f	56.53 c	127.46 cd	12.60 e	9.55 j	8.22 c	1.54 d
PRE 4b	3.93 efg	4.36 ef	12.50 g	0.25 f	56.53 c	130.66 b	12.56 f	9.59 ij	8.12 d	1.68 c
PRE 4c	3.96 bc	4.48 d	12.76 f	0.29 e	58.66 b	133.86 a	12.52 g	9.62 i	8.02 e	1.56 d
POST 1a	3.96 bdc	4.33 fgh	13.44 bc	0.29 e	40.53 gh	104.53 k	12.62 d	10.35 c	7.22 f	1.30 ef
POST 1b	3.96 bcd	4.25 ij	13.48 b	0.31 cd	40.00 h	100.80 l	12.58 e	10.34 cd	7.17 g	1.25 f
POST 1c	3.94 cde	4.63 b	13.83 a	0.34 b	36.26 i	90.66 n	12.59 e	10.32 d	7.22 f	1.32 e
POST 4a	3.97 b	4.34 fg	13.48 b	0.30 de	36.26 i	96.53 m	14.62 b	11.23 b	9.37 a	2.96 a
POST 4b	3.98 b	4.32 gh	13.39 bc	0.31 cd	41.60 g	101.86 l	14.43 c	11.21 b	9.08 b	2.77 b
POST 4c	3.94 def	4.56 b	13.45 bc	0.35 b	41.60 g	82.133 o	14.75 a	11.62 a	9.09 b	2.75 b

Data are means of triplicate measurements, except for volatile acidity, which was measured in duplicate. Means within a column followed by different letters are significantly different (one-way ANOVA, Tukey’s HSD post hoc, *p* < 0.05). The relative standard deviation of the technical replicates was no more than 4% for each wine. a*, b* expressing the green‒red and blue‒yellow color components, respectively. Prefixes: PRE = *GL* extracts added prior to fermentation, POST = *GL* extracts added after the fermentation process.

**Table 2:** Concentration of volatile compounds (μg/L) in control and red wines containing *GL* added pre- or post-fermentation in 28 L ferments.

Compound	Control	PRE 1	PRE 2	PRE 4	POST 1	POST 4	Sig	Aroma Detection Threshold (μg/L)
ethyl acetate	3793.6 ± 2330.8b	7018.5 ± 604.1ab	8252.6 ± 1258.8a	7755.0 ± 998.4a	7374.0 ± 1810.3ab	8016.4 ± 561.9a	0.021	15,000 ** a
ethyl butanoate	26.1 ± 4.6	23.9 ± 2.2	25.9 ± 2.1	26.3 ± 1.3	29.4 ± 2.2	27.9 ± 2.8	0.292	20 ** a
ethyl-2-methylbutanoate	0.5 ± 0.0	0.5 ± 0.0	0.5 ± 0.0	0.5 ± 0.1	0.6 ± 0.1	0.5 ± 0.0	0.572	1 ** a
ethyl 3-methylbutanoate	0.6 ± 0.1	0.6 ± 0.1	0.7 ± 0.1	0.7 ± 0.1	0.7 ± 0.1	0.7 ± 0.0	0.653	3 ** a
3-methylbutyl acetate	614.1 ± 28.3	609.5 ± 124.1	636.6 ± 37.5	502.2 ± 6.9	563.3 ± 69.0	539.0 ± 41.9	0.156	30 ** a
ethyl hexanoate	60.3 ± 3.2	65.6 ± 3.2	60.8 ± 2.0	58.0 ± 6.3	66.2 ± 10.4	58.0 ± 5.6	0.381	14 ** b
hexyl acetate	32.3 ± 2.4	30.8 ± 5.6	29.0 ± 6.3	26.5 ± 3.6	29.9 ± 7.4	25.7 ± 2.7	0.603	670 ** a
ethyl lactate	5186.6 ± 494.4	5165.3 ± 551.4	5637.1 ± 565.7	5458.7 ± 1000.1	5742.9 ± 1164.8	5776.8 ± 366.6	0.845	146,000 ** a
ethyl octanoate	15.7 ± 0.9	15.8 ± 1.0	16.8 ± 1.6	18.0 ± 0.2	16.5 ± 1.8	15.8 ± 1.2	0.235	20 ** b
ethyl decanoate	28.5 ± 4.5	33.2 ± 3.9	41.2 ± 10.0	46.8 ± 6.2	35.3 ± 10.9	31.8 ± 6.2	0.093	200 ** b
diethyl succinate	1.4 ± 0.0	1.6 ± 0.0	12.2 ± 1.9	19.0 ± 13.6	10.8 ± 7.4	11.0 ± 3.3	0.052	1,250,000 ** a
2-phenylethyl acetate	21.2 ± 1.6	21.2 ± 5.1	20.8 ± 4.3	20.8 ± 3.2	16.4 ± 1.7	17.0 ± 1.5	0.277	250 ** a
1-propanol	9029.4 ± 732.8	8357.8 ± 570.6	7802.2 ± 770.4	8692.7 ± 616.2	9394.5 ± 277.2	9215.7 ± 589.7	0.066	500 ** b
2-methyl-1-propanol	2362.3 ± 161.8	2258.3 ± 231.4	2190.4 ± 24.5	2102.6 ± 46.6	2083.8 ± 57.4	2136.8 ± 30.3	0.107	40,000 ** b
1-butanol	64.3 ± 10.5	60.3 ± 11.2	67.3 ± 9.6	50.8 ± 8.5	71.5 ± 8.1	61.1 ± 13.3	0.29	150,000 * a
3-methyl-1-butanol	17185.2 ± 765.4	16411.5 ± 351.2	17774.1 ± 515.1	16513.6 ± 2882.3	17612.4 ± 256.5	16733.9 ± 560.0	0.69	30,000 ** a
1-hexanol	199.5 ± 13.8	194.7 ± 24.7	199.8 ± 14.9	193.7 ± 14.6	209.7 ± 7.0	211.1 ± 4.3	0.598	8000 ** a
Linalool	6.2 ± 0.6	6.7 ± 0.7	6.7 ± 0.3	7.0 ± 1.2	7.6 ± 0.9	6.8 ± 0.4	0.427	15 ** a
1-octanol	2.4 b ± 0.1	2.4 ± 0.2ab	2.6 ± 0.1ab	2.8 ± 0.2a	2.6 ± 0.0ab	2.6 ± 0.1ab	**0.041**	0.7 ** a
α-terpineol	5.0 ± 0.4	5.2 ± 0.1	5.6 ± 0.8	6.1 ± 0.9	5.4 ± 0.3	5.1 ± 0.5	0.267	250 ** b
benzyl alcohol	178.3 ± 18.7	179.5 ± 23.3	173.8 ± 22.7	166.1 ± 18.4	178.2 ± 11.7	159.9 ± 8.9	0.715	200,000 *** a
2-phenylethanol	1443.4 ± 123.0b	1587 ± 180.3ab	1545.8 ± 250.5b	1985.5 ± 162.7a	1356.9 ± 21.6b	1354.5 ± 26.4b	**0.002**	14,000 ** b
Limonene	0.4 ± 0.0b	0.5 ± 0.0a	0.6 ± 0.0a	0.6 ± 0.0a	0.6 ± 0.1a	0.6 ± 0.1a	**0.034**	15 ** a
Nonanal	2.8 ± 0.0	2.8 ± 0.0	2.8 ± 0.1	2.9 ± 0.1	2.9 ± 0.1	2.8 ± 0.1	0.461	2.5 ** a
3-methylbutanoic acid	45.6 ± 0.6bc	44.1 ± 2.2c	47.3 ± 0.7bc	50.7 ± 1.1a	47.5 ± 0.7ab	47.4 ± 1.2bc	**0.001**	33 * a
hexanoic acid	702.9 ± 29.9c	788.8 ± 13.1b	849.5 ± 46.4ab	887.5 ± 12.0a	779.1 ± 43.6bc	848.0 ± 4.8ab	**<0.001**	420 ** b
octanoic acid	1448.5 ± 87.3a	1392.1 ± 185.5a	1301.6 ± 112.0ab	1240.0 ± 67.6ab	1072.4 ± 71.6b	1181.0 ± 48.5ab	**0.009**	500 ** b
decanoic acid	375.7 ± 33.1a	362.5 ± 13.2a	330.7 ± 28.0abc	341.0 ± 13.1ab	293.3 ± 24.7bc	271.4 ± 26.2c	**0.001**	1000 ** b
β-damascenone	24.1 ± 7.4	20.7 ± 6.2	28.7 ± 2.9	21.8 ± 5.2	22.9 ± 4.0	25.7 ± 6.3	0.561	0.05 ** a

Mean values ± standard deviation of the three fermentation replicates. Bolded *p*-values indicate significant differences based on one-way ANOVA. Lower-case letters indicate significant differences between samples based on LSD post hoc comparison, *p* < 0.05). Prefixes: PRE = *GL* extracts added prior to fermentation, POST = *GL* extracts added after fermentation process. * Refers to Mayr et al. [47], ** Refers to Wang et al. [20], *** Refers to Zhao et al. [48]. Thresholds were reported for aqueous ethanol (a) and wine matrix (b).

## Supplementary Materials

The following are available online at https://www.mdpi.com/2304-8158/8/11/538/s1, Figure S1: Shiraz winemaking process under specific conditions in the 28 L winemaking experiment, Figure S2: Weighted regression coefficients of both aroma and flavor of red fruit (A and B), dried fruit (C and D) and mushrooms (E and F) notes, Table S1: Brix values (°) measured by refractometer from the beginning to the end of small-scale fermentation of chemically defined grape juice media (CDGJM) and red grape juice (RGJ), Table S2: Impact of *GL* on liking, perceived sensory attribute overall intensity and CATA ratings from benchtop evaluations of commercial Shiraz wine spiked with *GL* (panel n = 32) and medium-scale pre-fermentation *GL* wines (panel n = 11) and Table S3: Significant sensory attribute intensity means of *GL* wines (from 28 L ferments) generated by RATA panel.
