# Supplementary material for: Volatile Composition and Sensory Profiles of a Shiraz Wine Product Made with Pre- and Post-Fermentation Additions of Ganoderma lucidum Extract
Source: Foods. 2019 Nov 1;8(11):538. doi: 10.3390/foods8110538 (PMC6915547; doi:10.3390/foods8110538)
Supplement: Supplementary file 1 [file foods-08-00538-s001.pdf]

SUPPLEMENTARY INFORMATION FOR

Volatile composition and sensory profiles of a Shiraz wine product made with pre- and post-fermentation additions of *Ganoderma lucidum* extract

Anh N.H. Nguyen [1], Dimitra L. Capone [1,2], Trent E. Johnson [1], David W. Jeffery [1,2], Lukas Danner [1] and Susan S.P. Bastian [1,2,*]

[1] School of Agriculture, Food and Wine, The University of Adelaide, Waite Campus, PMB 1, Glen Osmond, SA, 5064, Australia; anhngochoang.nguyen@adelaide.edu.au; dimitra.capone@adelaide.edu.au; trent.johnson@adelaide.edu.au; david.jeffery@adelaide.edu.au; lukas.danner@adelaide.edu.au; sue.bastian@adelaide.edu.au.

[2] Australian Research Council Training Centre for Innovative Wine Production, The University of Adelaide, Waite Campus, PMB 1, Glen Osmond, SA, 5064, Australia.

* Correspondence: sue.bastian@adelaide.edu.au; Tel.:+61 883136647

TABLE OF CONTENTS

	Page
**Figure S1.** Shiraz winemaking process under specific conditions in the 28 L winemaking experiment.	S-2
**Figure S2.** Weighted regression coefficients of both aroma and flavor of red fruit (A and B), dried fruit (C and D) and mushrooms (E and F) notes.	S-3
**Table S1.** Brix values (°) measured by refractometer from the beginning to the end of small-scale fermentation of Chemically Defined Grape Juice Media (CDGJM) and Red Grape Juice (RGJ).	S-4
**Table S2.** Impact of *GL* on liking, perceived sensory attribute overall intensity and CATA ratings from benchtop evaluations of commercial Shiraz wine spiked with *GL* (panel n = 32) and medium-scale pre-fermentation *GL* wines (panel n = 11).	S-4
**Table S3.** Significant sensory attribute intensity means of *GL* wines (from 28 L ferments) generated by RATA panel.	S-5

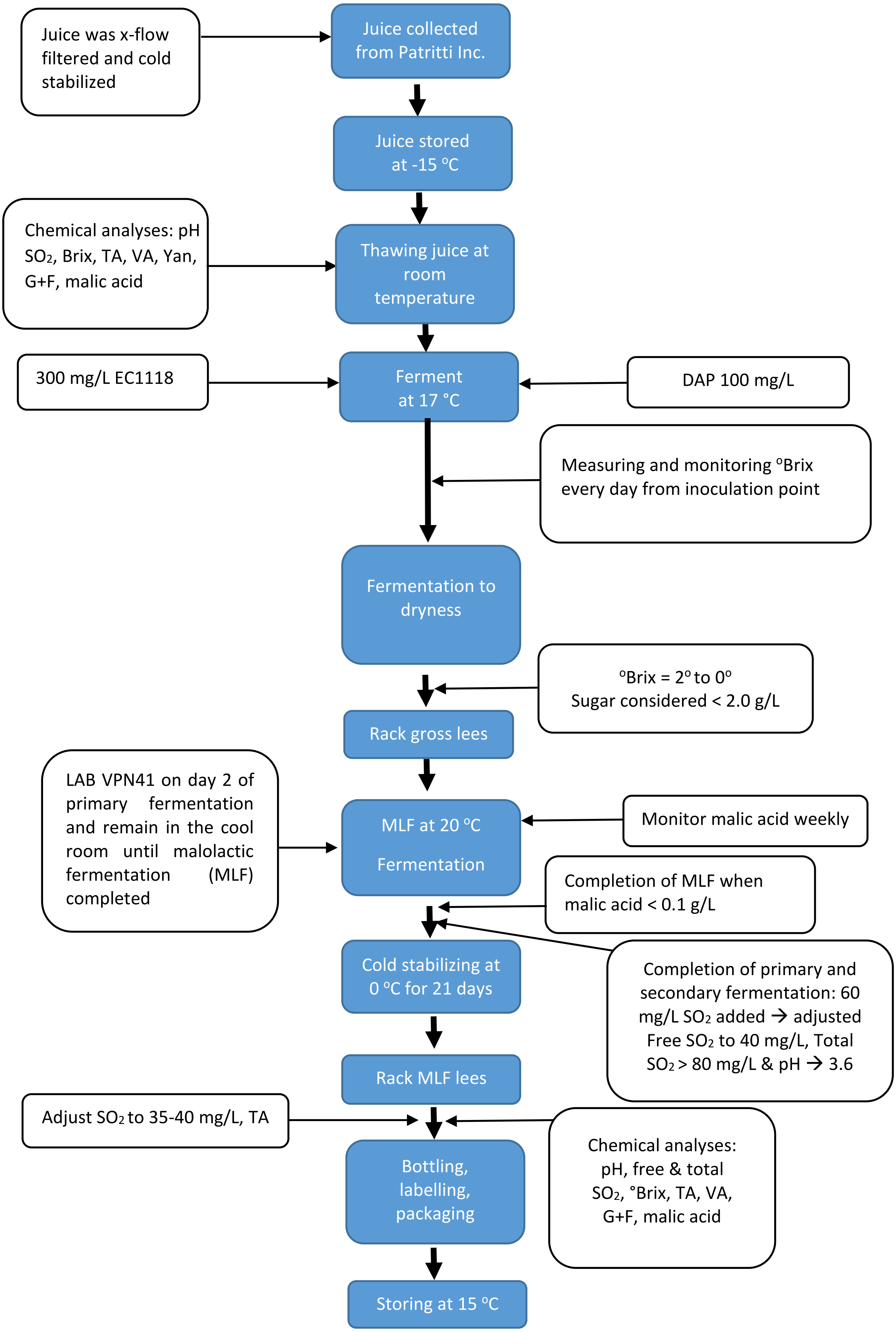

Figure S1. Shiraz winemaking process under specific conditions in the 28 L winemaking experiment.

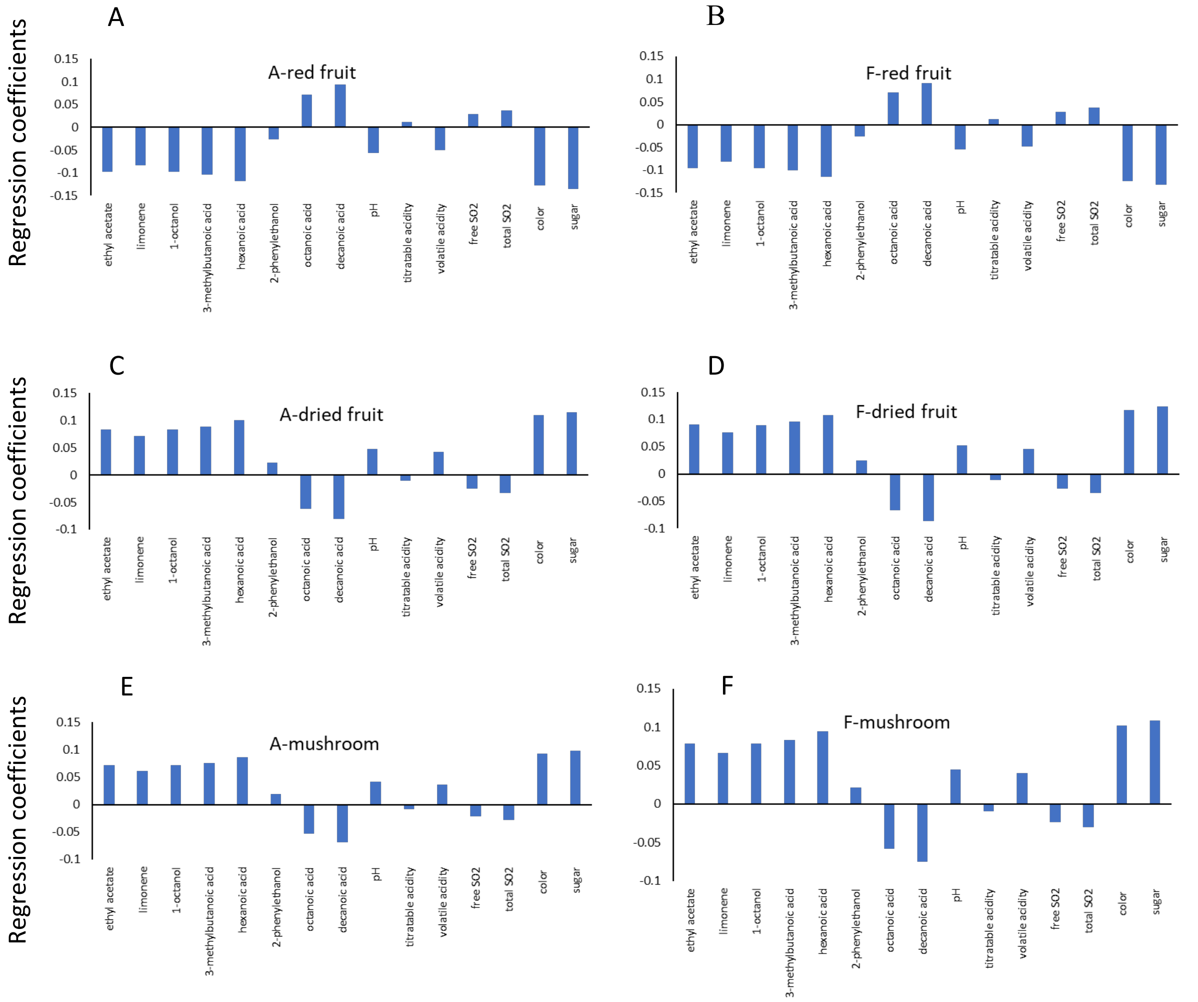

Figure S2. Weighted regression coefficients of both aroma and flavor of red fruit (A and B), dried fruit (C and D) and mushrooms (E and F) notes.

Table S1. Brix values (°) measured by refractometer from the beginning to the end of small-scale fermentation of Chemically Defined Grape Juice Media (CDGJM) and Red Grape Juice (RGJ).

	Day 0	Day 1	Day 2	Day 3	Day 4	Day 5	Day 6	Day 7	Day 8	Day 9
CDGJM-0	21.6 j	19.0 d	15.2 b	11.9 b	9.5 d	8.4 e	7.2 g	6.9 h	6.5 i	6.6 i
RGJ-0	23.6 f	19.9 b	15.9 a	13.0 a	10.8 b	9.6 c	8.3 e	8.4 e	8.3 f	8.2 ef
CDGJM-4.5	22.1 i	17.3 f	10.9 g	7.0 h	7.2 i	7.1 g	7.0 g	7.2 g	7.1 h	7.1 h
RGJ-4.5	23.8 e	19.6 c	14.6 c	11.2 c	9.1 e	8.4 e	8.2 e	8.3 e	8.2 f	8.1 f
CDGJM-9	22.6 h	17.3 f	11.1 fg	7.5 g	7.7 h	7.6 f	7.6 f	7.6 f	7.7 g	7.6 g
RGJ-9	24.1 d	19.6 c	13.7 d	10.5 d	8.9 f	8.7 d	8.6 d	8.6 d	8.6 e	8.4 e
CDGJM-18	23.4 g	17.4 f	11.2 fg	8.4 f	8.7 g	8.7 d	8.7 d	8.8 d	8.9 d	8.6 d
RGJ-18	24.6 b	19.6 c	13.5 de	10.4 d	9.5 d	9.5 c	9.3 c	9.3 c	9.2 c	9.3 c
CDGJM-36	24.3 c	17.7 e	11.4 f	9.7 e	9.9 c	9.8 b	9.8 b	9.8 b	9.7 b	9.8 b
RGJ-36	25.9 a	20.3 a	13.3 e	11.1 c	11.1 a	11.0 a	10.9 a	10.9 a	10.8 a	10.7 a

Means within a column followed by different letters are significantly different ($p < 0.05$)
Significance level at $p < 0.05$, data analyzed by one-way ANOVA, Fisher's LSD

Table S2. Impact of *GL* on liking, perceived sensory attribute overall intensity and CATA ratings from benchtop evaluations of commercial Shiraz wine spiked with *GL* (panel n = 32) and medium-scale pre-fermentation *GL* wines (panel n = 11).

Commercial Shiraz wine spiked with *GL*

		Overall intensity							CATA			
Wine	Liking	Aroma	Sweetness	Acidity	Hotness	Umami	Bitterness	Astringency	Earthy aroma*	Mushroom aroma*	Red berry aroma*	Oak aroma*
0	5.25 a	4.16 b	2.51 ab	4.45	4.25	2.93	4.03 b	4.06	22b	9c	53	47a
2.25	5.17 a	4.46 ab	2.96 ab	4.34	4.37	3.18	3.90 b	3.93	13b	22bc	53	22ab
4.5	4.72 ab	4.43 ab	3.00 a	4.31	4.40	3.34	4.40 b	4.06	25b	38abc	56	19b
6.75	4.48 ab	4.53 ab	2.28 b	4.18	4.12	3.59	4.56 b	3.93	34ab	44ab	31	19b
9.0	4.06 b	5.00 a	2.53 ab	4.06	4.09	3.56	5.28 a	4.18	56a	56a	28	28ab

Medium-scale pre-fermentation *GL* wines

		Overall intensity							CATA		
Wine	Liking	Aroma	Sweetness	Acidity	Hotness	Umami	Bitterness	Astringency	Floral flavor*	Tropical flavor*	Lychee flavor*
0	4.27	5.54	6.27 a	4.00 b	2.18 b	2.36	2.36 b	2.50	55a	73	73a
4.5	4.90	5.40	3.00 b	5.80 a	3.80 a	3.30	3.80 a	3.30	0b	36	9b
9.0	4.44	5.67	3.22 b	5.56 a	3.78 a	3.56	4.33 a	3.22	18ab	27	9b

Values for liking and aroma and palate attribute overall intensities are mean values. Means within a column followed by different letters are significantly different ($p < 0.05$) analyzed by one-way ANOVA, with Fisher's LSD. *Only significantly different attributes from the CATA evaluations shown as selection frequency percentage based on Cochran's Q tests are presented. Sheskin critical difference pairwise comparison was used to test significant differences between wine treatments and indicated by lower case letters.

Table S3. Significant sensory attribute intensity means of *GL* wines (from 28 L ferments) generated by RATA panel.

Sensory attributes	Control	POST 1	POST 4	PRE 1	PRE 2	PRE 4
Ap-red	4.2 a	4.0 b	3.9 b	4.0 b	4.1 b	3.7 c
Ap-brown	2.3 d	2.8 c	3.7 a	2.5 d	2.9 c	3.4 b
A-red fruit	3.7 a	3.3 b	2.9 c	3.6 a	3.5 ab	3.0 c
A-dried fruit	1.6 d	1.8 cd	2.2 a	1.6 d	1.9 bc	2.1 ab
A-jammy	2.0 ab	2.1 a	2.0 ab	1.7 b	2.0 a	2.0 ab
A-confectionery	2.9 ab	2.7 bc	2.1 e	3.0 a	2.5 cd	2.3 de
A-cooked vegetable	0.4 b	0.4 b	0.6 a	0.3 b	0.5 ab	0.7 a
A-earthy	0.7 c	0.8 bc	1.1 a	0.7 c	1.0 a	1.0 ab
A-floral	2.2 a	2.2 a	1.8 b	2.2 a	2.0 ab	1.7 b
A-mushroom	0.4 c	0.5 bc	0.8 a	0.4 c	0.6 ab	0.7 ab
A-leather	0.5 abc	0.5 bc	0.7 ab	0.4 c	0.6 ab	0.7 a
A-savory	0.7 b	0.7 b	1.2 a	0.7 b	1.0 a	1.1 a
A-spice	1.4 ab	1.3 b	1.5 a	1.4 ab	1.4 ab	1.2 b
A-toasty	0.8 cd	0.8 d	1.4 a	0.8 d	1.0 bc	1.1 b
A-woody	0.8 bc	0.9 bc	1.2 a	0.7 c	1.0 ab	1.0 ab
A-tobacco	0.5 c	0.6 bc	0.9 a	0.6 bc	0.6 bc	0.7 b
T-bitter	3.0 b	3.2 b	3.6 a	3.1 b	3.5 a	3.7 a
T-sweet	2.7 a	2.6 a	2.5 ab	2.5 ab	2.7 a	2.4 b
T-sour	2.7 b	2.8 ab	2.8 ab	2.9 ab	2.7 b	3.0 a
F-dark fruit	1.4 bc	1.6 ab	1.8 a	1.4 c	1.6 abc	1.6 abc
F-red fruit	3.5 ab	3.3 b	2.9 c	3.6 a	3.2 b	2.9 c
F-dried fruit	1.4 c	1.6 bc	2.0 a	1.4 a	1.7 ab	1.8 ab
F-jammy	1.7 a	1.9 a	1.9 a	1.4 b	1.9 a	1.6 ab
F-confectionery	2.4 a	2.2 ab	2.0 bc	2.3 a	2.2 abc	1.9 c
F-cook vegetable	0.4 ab	0.4 ab	0.5 a	0.3 b	0.5 ab	0.5 ab
F-earthy	0.7 cb	0.7 bcd	1.0 a	0.7 d	0.9 ab	0.9 abc
F-floral	1.9 a	1.8 a	1.5 b	1.8 a	1.8 a	1.5 b
F-mushroom	0.4 c	0.4 bc	0.7 a	0.4 bc	0.6 ab	0.6 a
F-green capsicum	0.5 ab	0.4 b	0.6 ab	0.5 ab	0.5 b	0.6 a
F-herbaceous	1.1 ab	0.9 b	1.1 ab	1.0 ab	1.0 ab	1.2 a
F-leather	0.4 bc	0.5 abc	0.7 a	0.4 c	0.6 ab	0.7 a
F-pepper	0.7 ab	0.6 b	0.8 ab	0.6 b	0.8 a	0.7 ab
F-savory	0.6 c	0.8 bc	1.0 ab	0.7 c	1.1 a	1.0 ab
F-spice	1.4 bc	1.4 bc	1.6 a	1.3 c	1.6 ab	1.4 abc
F-toasty	0.7 d	0.9 bcd	1.1 ab	0.8 cd	1.0 abc	1.2 a
F-woody	0.8 c	0.9 c	1.2 a	0.9 bc	0.9 bc	1.1 ab
F-tobacco	0.5 b	0.6 b	0.9 a	0.5 b	0.8 a	0.8 a
M-body	3.3 b	3.4 ab	3.5 a	3.3 ab	3.4 ab	3.3 ab
M-alcohol/heat	3.7 ab	3.8 ab	3.9 a	3.7 b	3.9 a	3.9 a
M-astringency	2.6 bc	2.8 ab	2.8 ab	2.5 c	2.7 ab	2.8 a
M-smoothness	3.5 a	3.3 abc	3.2 bc	3.4 ab	3.3 abc	3.1 c
M-roughness	2.4 bc	2.6 ab	2.6 ab	2.3 c	2.5 abc	2.7 a
FL-fruit	3.5 a	3.6 a	3.2 c	3.4 ab	3.5 ab	3.3 bc
FL-non-fruit	3.5 cd	3.5 bcd	3.7 ab	3.4 d	3.7 abc	3.8 a

Means within a row followed by different letters are significantly different. Significance level at $p < 0.05$, data analyzed by one-way ANOVA, Fisher's LSD. Prefix A- = aroma attribute, T- = taste; F- = flavor attribute, M- = mouthfeel, Ap- = appearance, FL- = aftertaste (fruit and non-fruit). Prefix PRE = *GL* added prior to fermentation (PRE 1, PRE 2 and PRE 4) and POST = *GL* added after fermentation (POST 1 and POST 4).
